# Dynamic Insulin Basal Needs Estimation and Parameters Adjustment in Type 1 Diabetes

**DOI:** 10.3390/s21155226

**Published:** 2021-08-02

**Authors:** Jesús Berián, Ignacio Bravo, Alfredo Gardel-Vicente, José-Luis Lázaro-Galilea, Mercedes Rigla

**Affiliations:** Campus Universitario s/n, Polytechnic School, University of Alcala, Alcala de Henares, 28805 Madrid, Spain; ignacio.bravo@uah.es (I.B.); alfredo.gardel@uah.es (A.G.-V.); josel.lazaro@uah.es (J.-L.L.-G.); mrigla@tauli.cat (M.R.)

**Keywords:** diabetes, closed-loop, insulin control, basal needs, artificial pancreas

## Abstract

Technology advances have made possible improvements such as Continuous Glucose Monitors, giving the patient a glucose reading every few minutes, or insulin pumps, allowing more personalized therapies. With the increasing number of available closed-loop systems, new challenges appear regarding algorithms and functionalities. Several of the analysed systems in this paper try to adapt to changes in some patients’ conditions and, in several of these systems, other variables such as basal needs are considered fixed from day to day to simplify the control problem. Therefore, these systems require a correct adjustment of the basal needs profile which becomes crucial to obtain good results. In this paper a novel approach tries to dynamically determine the insulin basal needs of the patient and use this information within a closed-loop algorithm, allowing the system to dynamically adjust in situations of illness, exercise, high-fat-content meals or even partially blocked infusion sites and avoiding the need for setting a basal profile that approximately matches the basal needs of the patient. The insulin sensitivity factor and the glycemic target are also dynamically modified according to the situation of the patient. Basal insulin needs are dynamically determined through linear regression via the decomposition of previously dosed insulin and its effect on the patient’s glycemia. Using the obtained value as basal insulin needs and other mechanisms such as basal needs modification through its trend, ISF and glycemic targets modification and low-glucose-suspend threshold, the safety of the algorithm is improved. The dynamic basal insulin needs determination was successfully included in a closed-loop control algorithm and was simulated on 30 virtual patients (10 adults, 10 adolescent and 10 children) using an open-source python implementation of the FDA-approved (Food and Drug Administration) UVa (University of Virginia)/Padova Simulator. Simulations showed that the proposed system dynamically determines the basal needs and can adapt to a partial blockage of the insulin infusion, obtaining similar results in terms of time in range to the case in which no blockage was simulated. The proposed algorithm can be incorporated to other current closed-loop control algorithms to directly estimate the patient’s basal insulin needs or as a monitoring channel to detect situations in which basal needs may differ from the expected ones.

## 1. Introduction

Through the years, people living with type-1 diabetes have been using different types of therapies and insulins [1]; beginning with the first insulins obtained from pigs and the first human-analogue, going through regular, NPH (Neutral Protamine Hagedorn) and long-acting insulin along with tight meal schedules, to nowadays in which we have fast-acting insulins and pump therapy with Continuous Glucose Monitoring (CGM) that allows the patient to achieve much better results and have a more normal life in terms of meals [2]. In the past, insulin therapy and meal schedules could not be modified easily because the long or intermediate effect of insulin had to match the patient’s insulin needs, and food has a significant impact on these needs. With the appearance of fast and ultra-fast short-acting insulin, therapies evolved into what is called basal-bolus therapy: Basal insulin, or background insulin, is the insulin dosed to the patient to keep his/her glucose levels stable during periods of fasting. The patient uses long-acting insulin to cover his/her basal needs and uses fast insulin to counteract the effect of meals. This type of therapy allows the patient to dynamically adjust the amount of insulin needed. In the last 20 years, there has been incremental improvement in terms of technology for diabetes: disposable insulin pens, improved glucometers, insulin pumps and continuous glucose monitoring technology (CGM) among them. Nowadays, the use of closed-loop systems [3,4,5] with continuous glucose monitors and insulin pump therapy with fast or ultra-fast insulin provide a more flexible way to control diabetes.

Studies show that basal needs change depending on factors such as the time of the day, exercise [6], stress, illnesses [7], cholesterol or age [8]. Most commercial systems assume that basal needs for a specific patient are the same from day to day and, therefore, only one basal profile is used and refined using past days’ information. The patient should activate parameter modifiers for exercise or illness situations that compensate for those effects and still have safe control. Other recent studies [9] take a different approach to this problem and, instead of separately modeling the insulin basal needs for the patient, the control is performed using PID techniques which treat the problem.

In this paper, we propose an algorithm that has been designed to dynamically evaluate the patient’s basal needs so that a closed-loop control algorithm can take advantage of this information to better adjust therapy. This solution has several advantages, such as how the patient does not need to adjust the basal profile. The basal profile is semi-automatically adjusted for each situation. The absorption of low glycemic index meals can be compensated as an increase in the basal needs. The proposed system is described as a semi-automatic system since there are still a set of parameters that need to be manually set to obtain correct basal need determination. Some of these parameters are insulin sensitivities and the action curve for the type of insulin being delivered (usually defined by the type of insulin and the duration). Other recent studies have shown efficient methods to estimate some of these parameters, such as the insulin sensitivities [10], and using both methods simultaneously could lead into a fully automated system that adjusts the insulin basal needs of the patient.

In order to evaluate the results offered by this new method for determining the patient’s basal needs, as there is no direct way to measure them, some simulation scenarios have been created. In these simulations, using a simple control algorithm as a basis, basal needs have been externally modified and the results from the controller in each case can be compared.

The proposed method uses these pre-set parameters to create a model of how insulin should affect the patient’s glycemia and, from there, obtain the corresponding basal needs. The derived basal needs are later used in a closed-loop control to determine the appropriate action to be taken [11].

## 2. Methods

### 2.1. Proposed Algorithm to Dynamically Determine Basal Insulin Needs in Closed Loop 

The patient’s basal insulin needs do not only vary from day to day but can also change from minute to minute depending on many parameters as exercise, illness, food intake, etc. The proposed algorithm tries to dynamically determine the current basal insulin needs to address this problem, giving the ability to adjust to the patient’s current situation. 

Before describing the algorithm, some concepts will be reviewed in this section as guidance.

### 2.2. Bolus Decomposition

The effect of one unit of insulin can take up to several hours to disappear and, therefore, it must be considered. The effect of every bolus of insulin can be translated or decomposed into endogenous-equivalent instantaneous boluses every few minutes, according to the insulin’s response curve. 

An instantaneous bolus (iBolus) is defined as the amount of insulin that, according to the insulin’s response curve, is acting on the patient at a specific moment in time. Instantaneous Insulin-On-Board (iIOB) is defined as the sum of all the instantaneous boluses that occur at the same time coming from different boluses.

For example, assuming the patient has an Active Insulin Time (AIT) of 4 h (for the type of insulin being used at that moment), if the patient delivered a bolus of 0.5 unit two hours ago and another two units bolus 1 one hour ago, the instantaneous boluses that are causing effect can be calculated as:

- iBolus_1_ = 0.5 ∗ AIT_4h_ (2.0)
- iBolus_2_ = 2.0 ∗ AIT_4h_ (1.0)

**Figure 1:**
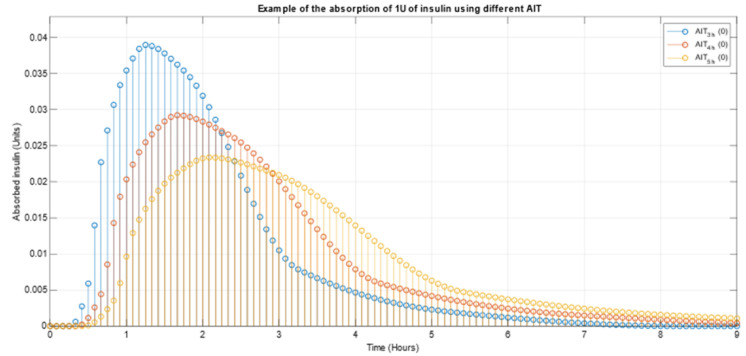
Example of the absorption of 1U of insulin using different AIT.

The AIT_4h_ function provides a time series of how one unit of insulin is absorbed, according to its absorption curve, when the bolus occurs some time prior to the current moment (specified as the parameter). The absorption curve must be determined according to the type of insulin being used by the patient. All elements in this time series are considered instantaneous boluses. In this same example, the iIOB for the patient at this precise moment would be equal to the sum of all the instantaneous boluses (iBolus_1_ and iBolus_2_ in the example). Figure 1 shows, in a graphical way, an example of how one unit of the same type of insulin gets absorbed in time depending on the AIT (blue = 3 h, red = 4 h and yellow = 5 h). 

**Figure 2:**
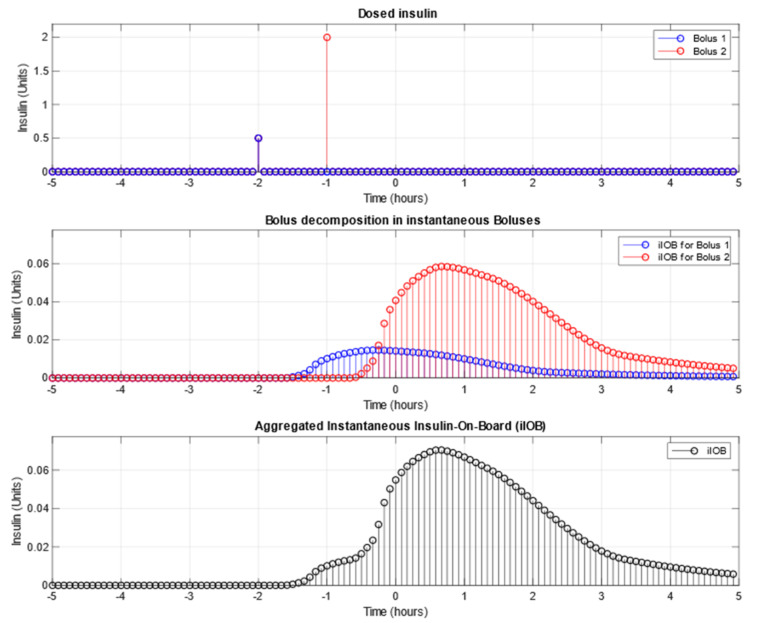
Example of iIOB decomposition for 2 boluses.

Figure 2 shows the example of how the two previous boluses, respectively represented in the “Dosed Insulin” subfigure as two Kronecker deltas in color blue (Bolus 1) and red (Bolus 2), are decomposed into instantaneous boluses in the “Bolus decomposition” subfigure and then, all effects are aggregated to obtain the instantaneous IOB or iIOB in the “Aggregated Instantaneous Insulin On Board” subfigure. 

In the first subfigure (Dosed Insulin), the two boluses are represented with their corresponding amount, 0.5 units for Bolus 1 and 2.0 units for Bolus 2, and their respective start time, 2 h ago for Bolus 1 and 1 h ago for Bolus 2. As can be seen in the bolus decomposition subfigure, the effect tail of Bolus 1 overlaps with Bolus 2: that is the reason why, once the iIOB in the bottom subfigure is calculated, more insulin effect is expected during the time associated with Bolus 2 (from −00:30 to 02:00 approximately). The peak effect caused only by Bolus 1 reaches 0.018 Units/5 min, while the peak effect caused by Bolus 2 and Bolus 1′s tail reaches 0.07 Units/5 min.

Insulin-On-Board (IOB) estimates all the insulin that still needs to be absorbed once the basal insulin has been subtracted, or that still needs to cause some effect. On the other hand, iIOB estimates the insulin that is causing effect at a specific time due to previously delivered insulin. IOB can be calculated as the sum of all the iIOBs from the current time to the future, subtracting the basal insulin present at each instantaneous bolus. 

Equation (1) mathematically describes the relationship between IOB and iIOB. IOB evaluated at time *t* is equal to the sum of all iIOBs from that moment on subtracting the basal needs.

(1) $${\mathrm{IOB}}_{t}=\sum_{n=t}^{\infty }\left({\mathrm{iIOB}}_{n}-\mathrm{Basal}\right)$$

Equation (1)—Relationship between IOB and iIOB.

### 2.3. Basal Insulin Needs and Insulin Sensitivity Factor Estimation

The Insulin Sensitivity Factor (ISF) is defined as the expected reduction in the patient’s glycemia caused by one unit of the dosed insulin. 

Basal needs are defined as the amount of insulin needed to keep the patient’s glycemia stable. The (un)stability of the glycemia can be calculated as the first derivative of the glucose levels reported by a continuous glucose sensor (derivative of sensor glucose value-dSGV). The insulin causing effect for every CGM data point can be calculated as the iIOB using the sampling period that corresponds with the CGM data (typically 5 min). For this iIOB to be correct, the algorithm needs to operate with the correct insulin’s response curve. As a simplification, the algorithm needs to know the insulin type and the Active Insulin Time for the patient. With this information, the algorithm calculates the equivalent response curve to be used.

Ideally, using the last iIOB-dSGV duples in a simple linear regression, basal insulin needs and the Insulin Sensitivity Factor (ISF) could be derived. The basal needs would be equivalent to the iIOB that makes dSGV be zero and the ISF would be the slope of the line resulting from the linear regression.

**Figure 3:**
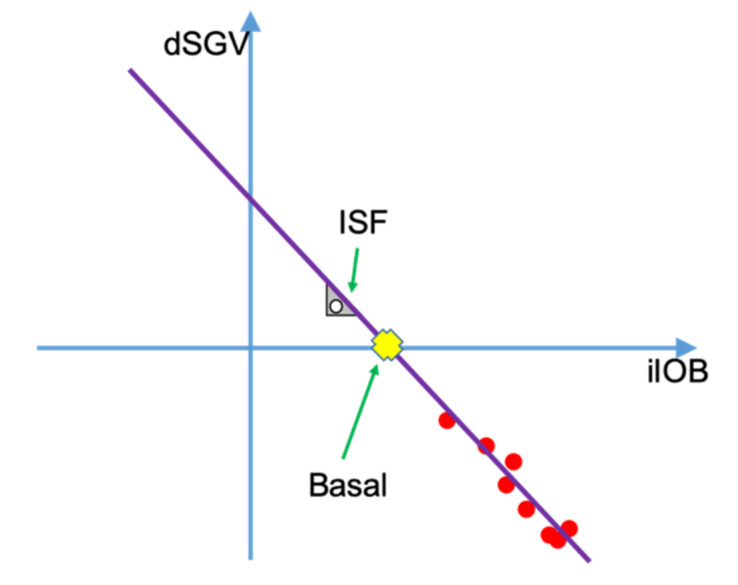
Relationship between dSGV and iIOB.

Figure 3 shows an example of the relationship between dSGV and iIOB. dSGV-iIOB duples have been represented using red dots. As can be expected, the greater the iIOB the more negative the dSGV, meaning that the more instant insulin is active the greater glycemic reduction. Through linear regression (purple line), the amount of instant insulin that zeros the dSGV (yellow cross), which can be also described as the amount of insulin that makes the patient glycemia stable (basal needs), can be calculated. The ISF can be calculated as the slope of the line resulting of the linear regression multiplied by minus one.

Equations (2)–(4) describe the method to calculate the basal needs and the ISF using classical least-squares linear regression, being *N* the number of tuples or data points used in the regression algorithm. From the relationship between iIOB and dSGV (Equation (2)), the ISF estimation (Equation (3)) and the basal needs estimation (Equation (4)) can be easily derived.

(2) dSGV=−ISF ×(iIOB−Basal)

Equation (2)—Relationship between iIOB and dSGV.

(3) $$\mathrm{ISF}=-1\times \frac{N\times \sum_{i=0}^{N-1}\left({\mathrm{dSGV}}_{i}\times {\mathrm{iIOB}}_{i}\right)-\sum_{i=0}^{N-1}{\mathrm{dSGV}}_{i}\times \sum_{i=0}^{N-1}{\mathrm{iIOB}}_{i}}{N\times \sum_{i=0}^{N-1}{\left({\mathrm{iIOB}}_{i}\right)}^{2}-{\left(\sum_{i=0}^{N-1}{\mathrm{iIOB}}_{i}\right)}^{2}}$$

Equation (3)—ISF estimation through least-squares linear regression.

(4) $$\mathrm{basal}=\frac{\sum_{i=0}^{N-1}{\mathrm{dSGV}}_{i}-\left(-\mathrm{ISF}\right)\times \sum_{i=0}^{N-1}{\mathrm{iIOB}}_{i}}{N\times \mathrm{ISF}}$$

Equation (4)—Basal needs estimation through least-squares linear regression.

A problem appears when basal needs rapidly change: using current technology the algorithm can obtain a sensor glucose value (SGV) every five minutes. For the linear regression to work properly, due to noise in the sensor readings, among other effects, the algorithm needs several data points. The more data points used, the slower the response of the algorithm. 

On the other hand, if not enough data points are used, and the used ones are close to each other, the linear regression algorithm could obtain erratic results. If the number of data points is small, it may be difficult to detect the linear regression’s assumption violations [12]. Effects such as heteroscedasticity of variances or nonnormality can be difficult to detect. What is even more important is that it is difficult to know if the result matches the patient’s real response.

**Figure 4:**
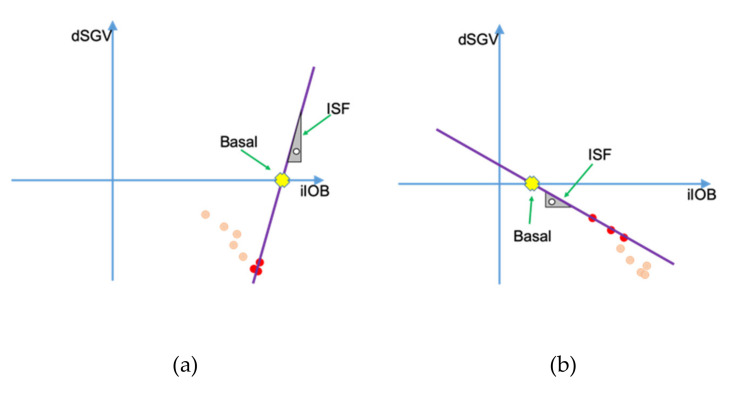
Example of erroneous results due to reduced data sets. (**a**) Using subset1 (**b**) Using subset2.

As can be seen in the examples of Figure 4a,b, in the absence of disturbances such as meals or exercise and only due to noise present in the glucose sensor readings, depending on the points used for the linear regression, results can vary dramatically. In Figure 4a, the results from the linear regression would give a much higher value for the basal needs and an illogical sensitivity: the change of sign in the slope would suggest that the patient’s glycemia would increase with an increase in the active insulin, which is incorrect. In Figure 4b, the chosen subset of data would lead to much lower basal needs than what the patient really needs and, therefore, to a decrease in the control results.

A rapid increase in the insulin basal needs, such as the increase that happens right after high glycemic index meals, could lead to situations in which the obtained duples may not correctly represent this relationship: basal needs from one duple to the next one may vary and, therefore, the result of the linear regression may offer incorrect results. This issue can be mitigated through any subset of the following procedures:

- Meal announcement: in hybrid closed-loop systems meals should be announced by the patient. If a meal is announced, the results from this algorithm should be discarded.
- Meal detection: some algorithms use rapid increments in glucose readings to detect meals. As with the meal announcement, if a meal is detected, results from the algorithm should be discarded.
- Fixing the insulin sensitivity factor (ISF) to mitigate the error.

Basal insulin needs change rapidly depending on factors such as food, exercise, infusion set state or insulin degradation, but insulin sensitivity is less affected by those parameters: guidelines for bolus calculator settings even consider it a constant value based on the patient’s total daily dose [13]. To avoid situations such as the ones shown in Figure 4a,b, and having into account that the ISF, under normal circumstances, is less affected by external factors than other parameters, the basal needs determination algorithm tries to mitigate this problem by fixing the ISF for the patient and, along with it, the slope of the resulting line. Insulin sensitivity is obtained from a set of predefined insulin sensitivities for every time of the day. They are calculated with classical methods out of the scope of this article [14].

Using predefined insulin sensitivity factors (ISF), the problem of estimating the right value for the insulin basal needs is reduced to finding the line that best fits the data points using the predefined ISF (slope). Basal needs estimations are still affected by the sensor’s noise, but the effect is greatly minimized since the slope is no longer affected by the data set.

**Figure 5:**
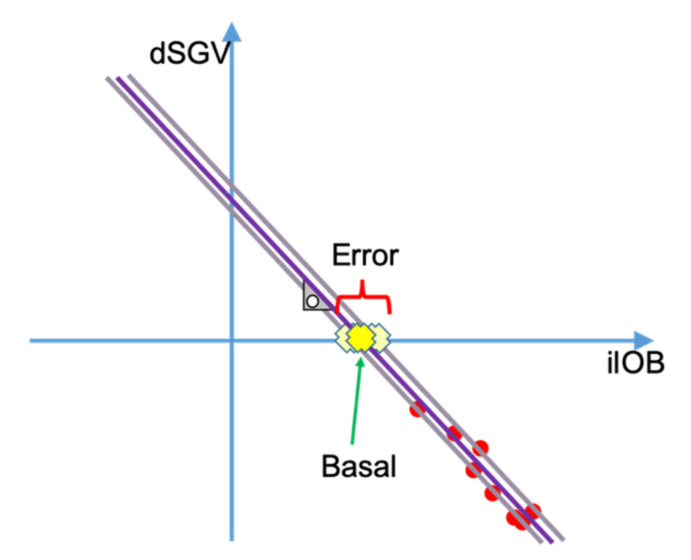
Basal needs estimation using fixed ISF.

Figure 5 shows in an example how, by fixing the insulin sensitivity factor, the error is reduced and fewer tuples can be used to calculate the basal needs. This simplification allows the algorithm to use fewer data points and respond faster to changes in insulin needs.

Equation (5) describes the method used to calculate the estimated basal needs (*eBasal*) using an ISF predefined by the patient (*ISF_predefined_*) using *N* tuples for the calculation.

(5) $$eBasal=\frac{\sum_{i=0}^{N-1}{\mathrm{dSGV}}_{i}-\left(-ISF_{predefined}\right)\times \sum_{i=0}^{N-1}{\mathrm{iIOB}}_{i}}{N\times ISF_{predefined}}$$

Equation (5)—Estimated basal needs with predefined ISF.

The problem of adjusting the ISF for the patient can also be solved applying methods such as the one described in [10], resulting in a fully automated determination system.

The proposed system falls into the category of single-hormone controllers [15], meaning that the controller only acts over the infusion of one hormone, which, in the case of the proposed system, is insulin. Other systems, called dual hormone, also control the infusion of a second hormone, typically glucagon. Glucagon has the opposite effect to insulin, rising glucose levels and allowing the controller to affect the patient’s glycemia in both ways, lowering and raising it as needed. When estimating basal needs for a single-hormone controller, the estimations must be conservative and safe for the patients since there is no mechanism to cancel the effects of already-dosed insulin. Data obtained from the continuous glucose monitor (CGM) has some delay over the actual blood glucose values, and this delay can vary from 10 to 20 min depending on several factors [16]. Any estimation performed using those data points will suffer from the same delay and, if not considered in situations when basal needs might be dropping, the controller could dose more insulin than needed, possibly causing a hypoglycemic event to the patient. 

To compensate the problem, the trend on the basal estimation is analyzed and used to estimate basal needs at that current moment. As the controller only uses insulin for the control, basal needs must be conservative: if basal needs are increasing, the trend will not be added to the result and, if basal needs are decreasing, the trend will be included to sooner reduce the amount of insulin being delivered. Therefore, the controller will keep the basal needs estimation as low and safe as possible.

Equation (6) shows how the trend in the estimated basal (*eBasal*) is calculated. The first derivative of the estimated basal needs determines how the estimated basal needs have been changing with time. Using only the last data point from the derivative would provide the controller with the fastest response to the change but, as data coming from the continuous glucose sensor may incorporate a significant amount of noise in the measurement, the insulin action curve may be slightly off; large fluctuations on the result are noticeable and impact the controller’s performance. Basal insulin needs do not rapidly change compared to current CGM’s sampling period, and the sampling period is short enough to allow some degree of filtering. Several tests were made and a simple average of the last 15 min of the first derivative showed good results, balancing response time and noise reduction. This window can be increased, incrementing the time that the algorithm needs to respond, or decreased, being more susceptible to changes due to noise. The variability of the signal to noise ratio in CGM systems depends on the state of the sensor, so 15 min was used as a midpoint for the tests.

(6) $$trend_{eBasal}=avg{\left(\;(eBasal_{last15}\right)}^{’})$$

Equation (6)—Trend in estimated basal.

The final estimated basal needs (Equation (7)) are calculated using the basal need estimation and its trend. Even though it might seem reasonable to compensate the CGM delay in the basal estimation, increasing the value for basal needs is not appropriate since we need to ensure safety; insulin cannot be removed from the body once dosed and the controller can always dose more insulin in the future if needed when the increase in basal needs has been confirmed. The same reasoning was used to do the opposite when a decrease in basal needs is detected: the controller can reduce as much insulin as the prediction suggests, reducing the risk of hypoglycemia, because it can be dosed later if the trend prediction turns out to be wrong. As a result, if the trend is equal or greater than zero, the estimation of the basal needs is not modified and becomes its final version. If the trend is lower than zero, meaning that the patient needs less insulin as time passes, basal needs are reduced to accommodate to this trend, calculating the expected decrease in the following 15 min (being this time the average delay in current CGM systems) and incorporating the effect to the estimated basal needs.

(7) $$eBasal_{final}\left(eBasal,trend_{eBasal}\right)=\{\begin{array}{c} eBasal+(trend_{eBasal}\times Period_{15min}),\;trend_{eBasal}<0\\ eBasal,\;trend_{eBasal}\geq 0 \end{array}$$

Equation (7)—Final Estimated Basal Needs.

### 2.4. Proposed Algorithm to Dynamically Adjust the Insulin Sensitivity Factor (ISF)

It is known that there is a relationship between the ISF and the patient’s glycemia: the ISF decreases when the patient’s glycemia increases [17]. To counteract this effect, another algorithm is included to dynamically modify the ISF according to the patient’s glycemia. This algorithm utilizes the configured ISF as the one determined for the patient when glycemia equals the target value (*ISF_target_*). As glycemia increases over the target, insulin sensitivity is linearly decreased according to a predefined ratio (*ratio_ISF_*). On the other side, when glycemia decreases below the target, the algorithm also decreases the insulin sensitivity to increase the needed correction and the safety of the system. As Equation (8) describes, these two last ISF modifications are equivalent to saying that the ISF estimation (*ISF_estimated_*) is equal to the configured ISF for when the patient is at the target glycemia minus the absolute glycemic error (*abs(error_glycemia_)*) multiplied by the ISF correction ratio. The glycemic error is the result from subtracting the glycemic target from the current glycemia.

(8) $$ISF_{estimated}=ISF_{target}-ratio_{ISF}\times abs(error_{glycemia})=ISF_{target}-ratio_{ISF}\times abs\left(sgv_{current}-sgv_{target}\right)$$

Equation (8)—Estimated Insulin Sensitivity Factor.

This adjustment is optional and can be disabled, setting the ratio to zero. The minimum value for the sensitivity (*ISF_min_*) is also set to avoid extreme results that could lead to undesired situations.

Equation (9) describes the way the estimated ISF (*ISF_estimated_*) is limited by the minimum ISF (*ISF_min_*) obtaining as a result the final ISF that will be used as in the closed-loop algorithm calculations (*ISF_final_*).

(9) $$ISF_{final}\left(ISF_{estimated},ISF_{min}\right)=\{\begin{array}{c} ISF_{min},\;ISF_{estimated}<ISF_{min}\\ ISF_{estimated},\;ISF_{estimated}\geq ISF_{min} \end{array}$$

Equation (9)—Final Insulin Sensitivity Factor.

**Figure 6:**
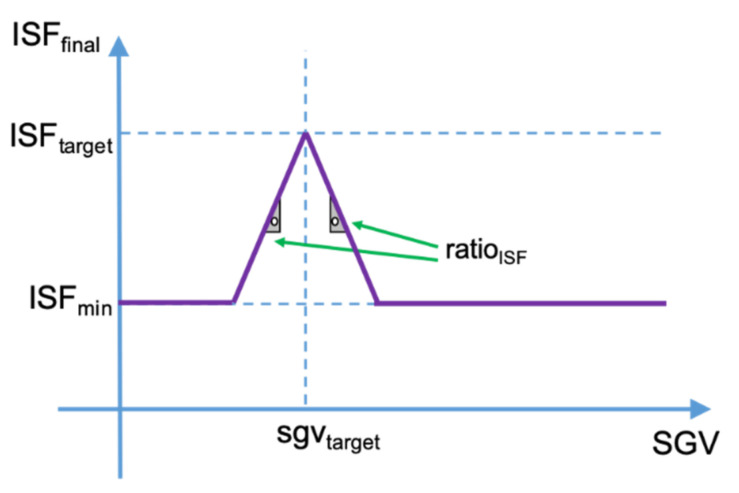
Graphical representation for the Final ISF calculation.

In Figure 6, a graphical representation of the relationship between the sensor glucose value (SGV) and the final ISF is provided as clarification.

### 2.5. Effects on Basal Estimation Due to Parameters Misconfiguration

The Insulin Action curve, defined by the absorption curve provided by the insulin manufacturer and the correction given by the patient as IAT, becomes a critical part on the estimation of the patient’s basal needs. 

The mismatch between the real absorption curve and the one being used by the algorithm will create areas in which the controller will estimate a higher iIOB than the real one, resulting in higher basal needs, and areas with lower iIOB than the real one, resulting in lower basal needs. 

**Figure 7:**
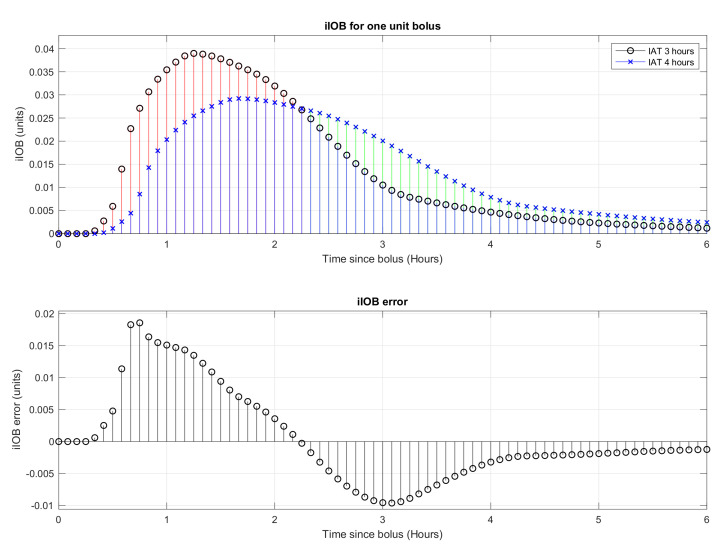
Example of error in estimated iIOB due to AIT misconfiguration.

Figure 7 shows an example in which the patient has configured an AIT of 3 h (plotted with circles in the iIOB subfigure) instead of 4 h (plotted with crosses in the iIOB subfigure) that would correspond to the real absorption curve for the patient. The two areas previously mentioned are plotted in red (higher estimated iIOB than the real one) and in green (lower estimated iIOB than the real one). The iIOB error subfigure shows the error in the iIOB estimation once the common part for both curves is subtracted (plotted in blue in the iIOB subfigure). These ups and downs in the iIOB error subfigure cause a ripple effect on the basal estimation and, therefore, on the control actuations and the final patient glycemia.

If the programmed insulin action curve does not match the insulin’s real response for the patient, oscillations in the patient’s glycemia can be expected. 

## 3. Simulation

The proposed algorithm was simulated, along with a simple closed-loop controller, using a free open-source version of the UVA/Padova simulator for type-1 diabetes named simglucose [18]. This simulator coded in Python provides models for a total of 30 patients: 10 children, 10 adolescents and 10 adults. 

The algorithm was coded in python, creating a Controller class, following the instructions from the simulator, implemented as was described in a previous article [11] and performing the following operations every simulation step:

1. Read configuration for the patient being simulated.
  - Type of insulin used and its response curve
  - Maximum Temporary Basal permitted
  - ISF_target_, ISF_ratio_ and ISF_min_
  - Glycemic target
  - Carbohydrates ratio
  - Prebolusing time and if it is enabled
  - Low-Threshold-Suspend glycemia
2. Only at the first iteration, the basal history that the controller uses to keep track of all its past actuations is filled with a predefined value, different for each patient. This has been done to avoid the border effect at the beginning of the simulation and be able to deliver basal insulin during the first cycles of operation. This is not necessary but allows the simulator to have valid data from the beginning.
3. Store the new CGM sensor data point and calculate its first derivative using the CGM history.
4. Calculate the iIOB corresponding to the administered basal insulin and the bolus insulin (treated separately).
5. Calculate the IOB.
6. Calculate the eventual glycemia of the patient (15 min trend prediction + the effect of the IOB).
7. Calculate the glycemic error as the difference between the eventual glycemia and the dynamic glycemic target for the patient.
8. Adjust the ISF according to the patient’s current glycemia and the glycemic error using Equations (8) and (9).
9. Calculate the patient’s current basal needs using Equations (5)–(7).
10. Calculate the insulin that will be added or subtracted to/from the basal needs in order to compensate the glycemic error. This amount of insulin is constrained to be between zero and the maximum temporary basal permitted for the patient.
11. If a meal will occur in the time defined as pre-bolusing time and pre-bolusing has been enabled, an insulin bolus is also delivered to counteract the effect of the coming carbohydrates. This bolus is calculated using the carbohydrate ratio configured for the patient.
12. Update all history vectors for the next simulation cycle.
13. Send Action to the simulator with the calculated basal insulin and the bolus insulin if needed.

### Glycemic Target and Low-Threshold-Suspend

Even though the glycemic target adjustment is not part of the proposed algorithm to determine the patient’s basal insulin needs, it was added to the closed loop controller [11] to minimize the risk of hypoglycemia. The proposed algorithm adapts the ISF according to patient glycemia, making the algorithm more aggressive as the glycemia increases. This glycemic target adjustment acts as a countermeasure to reduce the risk of hypoglycemia due to over-correction when ISF is lowered. 

The patient, or the healthcare professional, sets a glycemic target range defined by the lowest and highest glycemic values considered acceptable for that specific patient. This algorithm calculates the desired target as the mid-point of the target range, being that value of glycemia to be obtained while the patient glycemia is within the target range (Equation (10)).

(10) $$target_{desired}=\frac{target_{max}+target_{min}}{2}$$

Equation (10)—Desired glycemic target.

The lowest value is also used as low-threshold-suspend value, meaning that if the patient’s current or eventual glycemia is below that value, insulin infusion will be suspended independently of what predictions might suggest and an internal flag named Low-Threshold-Suspend flag (*LTS_flag_*) will be activated. Infusion is resumed when patient’s glycemia is safe and above the low-threshold-suspend value, but the Low-Threshold-Suspend flag will be kept active for 15 min to prevent the algorithm from being too aggressive after this event. This flag is used to temporarily set a higher target and reduce the abrupt response of the control loop in case the estimated basal rises due to correction of carbohydrate intake. 

During normal operation, the algorithm calculates the dynamic glycemic target (Equation (11)) as the mid-point between the patient’s current glycemia (sgv-Sensor *Glucose Value)* and the lowest value in the target range.

(11) $$target_{dynamic}=\frac{sgv+target_{min}}{2}$$

Equation (11)—Dynamic glycemic target.

The final glycemic target is constrained to be within the configured target range: the final target range will never be greater than the maximum target value or lower than the desired target. The *LTS_flag_* also acts as a temporary modifier that forces the final glycemic target to be the maximum value of the target range. This behavior is described in Equation (12).

(12) $$target_{final}\left(target_{dynamic},target_{desired},target_{max},LTS_{flag}\right)=\left\{\begin{array}{l} target_{max},\;\&LTS_{flag}=true\\ target_{desired},\;\&LTS_{flag}=false,\;target_{dynamic}\leq target_{desired}\\ target_{dynamic},\;LTS_{flag}=false,\;target_{desired}\;\leq target_{dynamic}\leq target_{max}\\ target_{max},\;LTS_{flag}=false,\;target_{dynamic}\geq target_{max} \end{array}\right.$$

Equation (12)—Final glycemic target determination.

**Figure 8:**
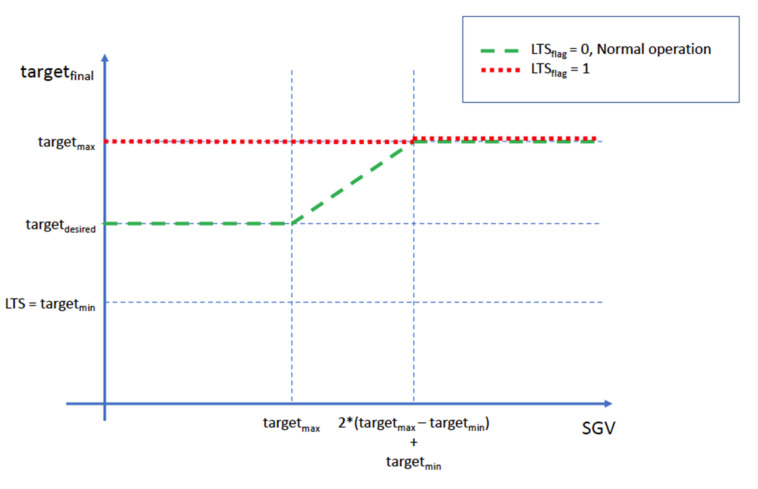
Relationship between the final glycemic target, the LTSflag and the Sensor Glucose Value.

In Figure 8 a graphical representation of the relationship between the sensor glucose value (SGV), the *LTS_flag_* and the resulting *target_final_* is shown as clarification.

## 4. Results

The proposed algorithm was simulated using simglucose [18] and the control algorithm described in a previous article [11] as a baseline. This simulator does not allow the inclusion of effects such as different types of food, glycemic indexes, exercise or hormonal disorders, but those effects will be studied and simulated in the future so that results can better match what a real scenario would be. To assess if the proposed algorithm is not only able to adapt to the initial patient’s basal insulin needs but to changes that could potentially occur at any time, the simulator was modified to include an infusion blockage effect. Using this blockage effect the simulation can control, with every simulation cycle, the amount of insulin that is commanded by the controller but not delivered to the patient. While insulin basal needs cannot be modified in the current state of the simulator, the infusion blockage offers similar effects from the point of view of the controller. 

Each patient requires different amounts of basal insulin, so, when insulin blockage is simulated, it needs to be adjusted accordingly so that results can be compared. To obtain meaningful results when insulin blockage is added to the simulation, the amount of insulin delivered to the patient after 12:00 is reduced by half of the amount that was previously determined as basal needs for that specific patient. This way, every patient is simulated with an equivalent increase of 50% in terms of basal insulin needs.

The simulations were grouped into batches so that all patients (30) could be simulated under the same scenarios at the same time. The duration of each simulation is 24 h, and their goal is to obtain evidence of the feasibility of the proposed algorithm to determine a value of basal insulin needs that can be used in a closed-loop algorithm and assess if this algorithm is also resistant to events that drastically modifies these needs (meals). The following batches were simulated:

- Batch 1: Simulations do not include meals or infusion blockage. The goal of this batch of simulations is to assess if the proposed algorithm can determine the basal insulin needs correctly in absence of any high impact disturbances such as meals.
- Batch 2: Simulations include four meals but no infusion blockage. The purpose of this batch of simulations is to assess if the controller keeps patients under control when meals are included.
- Batch 3: Simulations do not include meals, but infusion blockage occurs after 12:00. The intent of this batch of simulations is to assess if insulin basal needs are correctly determined even if insulin starts being blocked after some time (rising basal insulin needs from the controller’s point of view).
- Batch 4: Simulations include meals and infusion blockage after 12:00. The goal of this batch of simulations is to assess if the controller can successfully control the patient’s glycemia when meals occur while insulin is being blocked after some time. This batch of simulations offers the results that would be more like a real-world scenario.

In the first batch of simulations the goal was to assess if the proposed algorithm can correctly determine the basal needs for the patients. Simulations were executed for a 24 h slot of time and did not include carbohydrate intake. 

**Figure 9:**
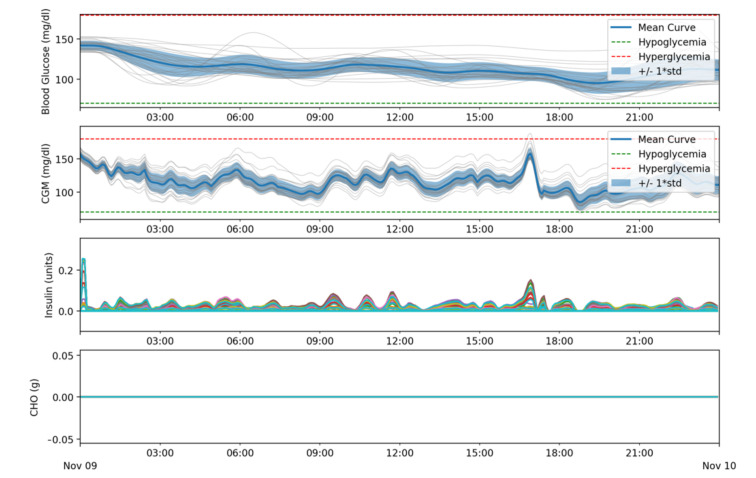
Glycemic and insulin delivery results for Batch 1.

As shown in Table 1, all patients were kept in the range 100% of the time. Figure 9 shows that, in all cases, the glycemia is kept in range and gradually corrected to be in the desired target (110 mg/dL). Certain visual correlation can be observed between the noise in the CGM data and the insulin delivery, but the algorithm responds to this, compensating afterwards.

Figure 9 shows the results obtained from the first batch of simulations. Results are shown in four different subfigures, using simulation time, measured in hours, as the *x*-axis:

1. Blood Glucose (mg/dL): shows the mean blood glucose values for all patients as well as the standard deviation. Grey lines correspond to the results obtained for each patient. The green and red lines delimit the target range (70–180 mg/dL).
2. CGM (mg/dL): shows the data received from the CGM sensor. Same colors and meanings as in the previous subfigure were used.
3. Insulin (Units): shows the amount of insulin that was dosed for each patient, represented each one by a different color. The simulator uses a time resolution of 1 min and, therefore, basal doses are represented in U/min units.
4. CHO (g): Shows the carbohydrate income for each patient. Each patient is represented with a different color but, as these simulations use the same meal schedule for all patients, only one plot can be seen. In a similar way to the Insulin subfigure, due to the simulator design, the carbohydrate absorption is measured in gr/min.

In the second batch of simulations, four meals were added to the simulation along with adding some pre-bolusing time between the insulin dosing for the meal and the meal intake, ranging from 10 to 60 min. Eating habits for children, adolescents, and adults might be different, so finding the right meal schedule for the simulation is not trivial. Evidence suggests that there is no ideal percentage of calories from carbohydrate, protein and fat for people with diabetes; meal plans should be individualized and reducing overall carbohydrate intake for individuals with diabetes has demonstrated the most evidence for improving glycemia [19]. Nevertheless, the goal of this second batch of simulations is not to obtain specific results in terms of Time-In-Range but to add high impact disturbances (meals) to see if the control is still acceptable and serve as reference to compare the results from following simulation batches. Therefore, meal contents in our simulations are not relevant.

The chosen schedule contains 140 g of fast-absorption carbohydrates that could be equivalent to a 2000 kcal schedule with a 28% content in carbohydrates or a 1500 kcal schedule with a 37% content in carbohydrate. The same meal schedule has been used for all patients and it is as follows:

- 07:00 → 56 g of carbohydrates
- 13:00 → 37 g of carbohydrates
- 17:00 → 9 g of carbohydrates
- 20:00 → 38 g of carbohydrates

As can be seen in Table 2, the time spent over 180 mg/dL and 250 mg/dL considerably increased, especially for adolescents and children. 

**Figure 10:**
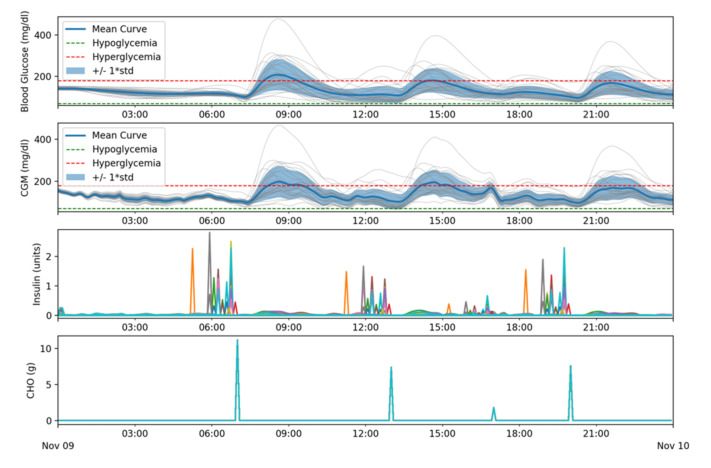
Glycemic and insulin delivery results for batch 2.

Figure 10 shows that these hyperglycemic events occurred during or after meals.

In a third batch of simulations, meals were removed and an increase in basal needs was simulated by deliberately reducing the amount of insulin the patient receives, simulating a partial blockage. Effects such as high-fat meals, illness or exercise cannot be simulated with the current state of the simulator but, as these effects can be simulated as modifications to the patient’s insulins basal needs at a specific time, simulating a partial blockage and being able to adapt to it should be enough to prove that, independently of the root cause, the system can adapt dynamically to changes in insulin basal needs. In this simulation, using the same set of parameters used in previous batches for the patients’ settings, the amount of basal insulin delivered to the patient after 12:00 is reduced by half of the amount that was set as pre-determined basal needs. This way every patient is simulated with an equivalent reduction in terms of relative needs.

**Figure 11:**
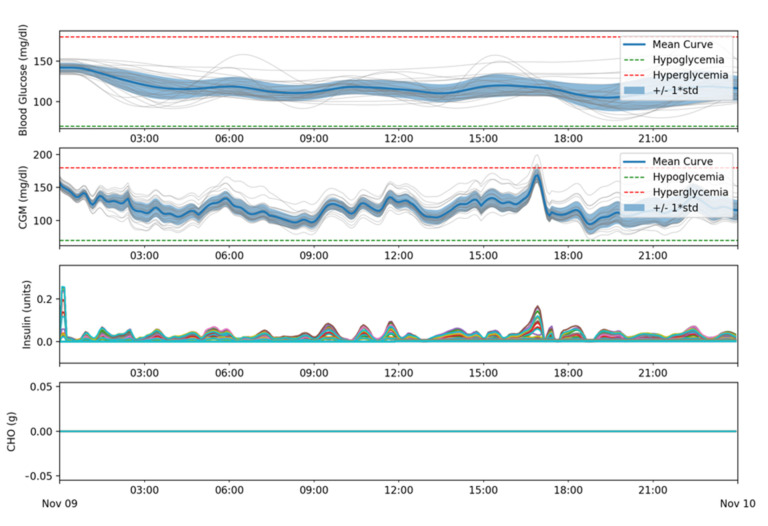
Glycemic and insulin delivery results for batch 3.

Table 3 shows a 100% time in range for all patients. Figure 11 shows similar results as Figure 9, as expected, showing that the change in basal needs is detected and dynamically compensated.

In a fourth batch of simulations meals were included as well as the partial insulin blockage after 12:00 for every patient. 

**Figure 12:**
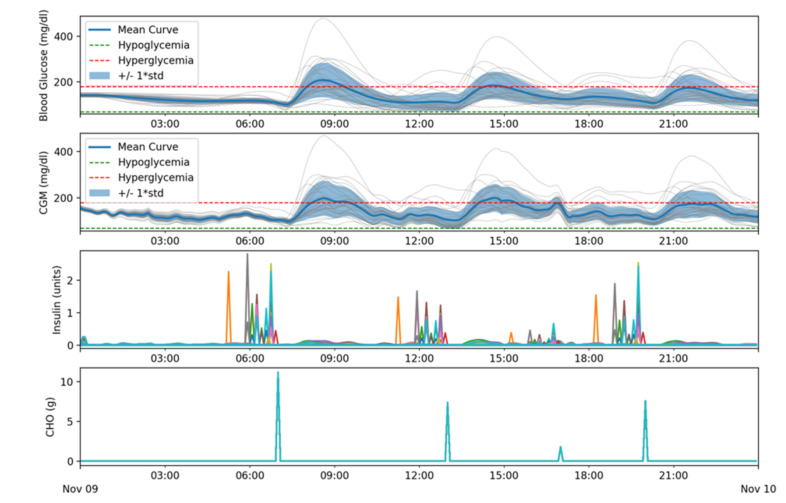
Glycemic and insulin delivery results for batch 4.

Table 4 shows, as expected, similar results to the ones in the second batch (see Table 2). Figure 12 shows similar results as Figure 10, meaning that the system successfully adjusted itself to the change in basal needs.

The Blood Glucose and the CGM subplots in Figure 12 show how, from 00:00 to 07:00, basal needs are correctly determined and all patients, in absence of meals, are kept in range. After each meal, shown in the CHO subplot, and their corresponding boluses, shown as spikes in the Insulin subplot, the patients’ glycemia tend to rise and is controlled afterwards taking it back in range. After 12:00 the insulin blockage is active and the algorithm continues working correctly.

Table 5 shows the insulin usage per patient, in insulin units, during simulation batches 1 and 3. These batches are the ones in which no meals are simulated, so that only basal needs are being evaluated, and the only difference is the insulin blockage during batch 3. As can be expected, Table 5 shows that the corresponding TDDs between 00:00 and 12:00 for both simulation batches offer the same results since both batches simulate the same conditions up to that point in time at which the blockage starts (12:00). On the other hand, global TDD (“TDD” columns) and TDD after 12:00 numbers are slightly higher for batch 3 than batch 1. These results are expected since batch 3 introduces the insulin blockage after 12:00. As was previously described, in batch 3 each patient suffered, from 12:00 to the end of the simulation (12 h in this case), an insulin blockage equivalent to half of their estimated basal during that period. The total amount of insulin blocked during simulation batch 3 can be observed in Table 5 in the “BLOCKAGE” column. The increment in TDD between batch 1 and batch 3 is shown in column “INCREMENT” and it can be observed that the increment is always lower than the blockage. This is caused mainly because, when the blockage occurs, basal insulin needs (from the algorithm’s point of view) almost instantaneously increase and the patient’s glycemia starts rising. This closed loop algorithm tries to reduce the risk of hypoglycemia raising the glycemic target when the patient’s glycemia increases, making the controller’s response slower and being conservative in terms of insulin usage. This behavior makes the patient stay longer at higher glycemic values and uses less insulin during the simulation.

**Figure 13:**
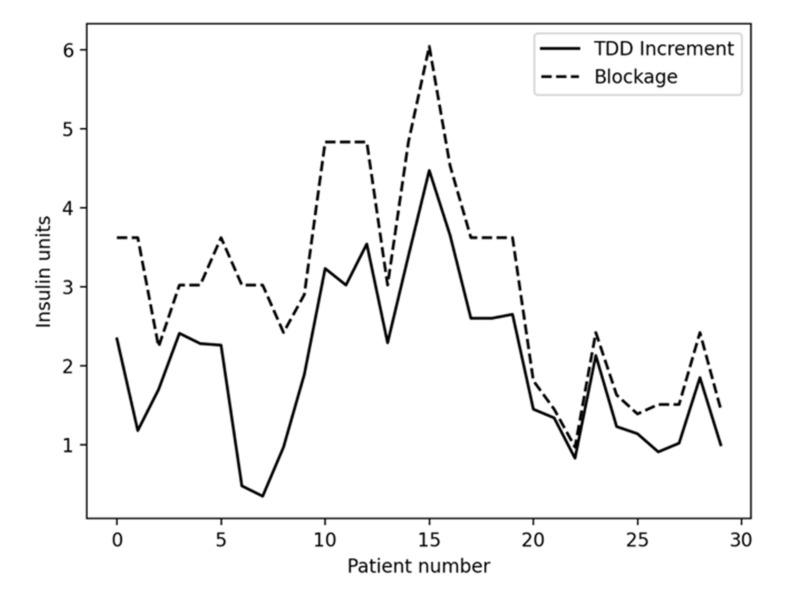
Insulin blockage vs. TDD increment between Batches 1 & 3.

Figure 13 shows in a graphical way the relationship between the amount of insulin being blocked for each patient and the corresponding TDD increment between Batch 1 and Batch 3. Although there is not a fixed relationship between the amount of blocked insulin and the increase in TDD due to the subtleties of the simulation and the controller, some correlation can be observed. The TDD increment is lower than the blockage because of the way the algorithm changes the glycemic target: when basal needs increase, as in the case of Batch 3, the glucose levels tend to rise. According to Equation (11), the glycemic target adapts to this situation, increasing its value to avoid being too aggressive, and the patient’s glycemia is kept a little higher for some time depending on the simulation conditions. 

## 5. Conclusions

Most used solutions rely on having a correct set of parameters that matches the patient’s needs (basal profiles, insulin sensitivity factors, etc.). These sets remain constant until the patient takes the action of modifying them to accommodate to his/her current situation. This algorithm reduces the number of parameters the patient needs to configure and dynamically adjusts to the patient state, obtaining acceptable in silico results in terms of control. This automation also reduces the number of tasks that the patient needs to take care of, making his/her life easier.

One limitation the proposed algorithm has is that the insulin response on the patient’s body needs to be characterized in advanced to obtain correct calculations. This characterization process has been performed using standard methods to determine ISF and the insulin action curve for the patient. As a next step, this algorithm could be improved by dynamically determining the ISF for the patient [10] as well as the insulin action curve, reducing even more the number of parameters to be configured in the system. There are several methods that could be explored to obtain these parameters; for instance, improvements in CGM technology to reduce noise in each measurement and/or increase the sampling rate allowing other types of filtering or using signal-processing techniques to match control-loop predictions with real data during fasting periods.

The proposed algorithm could, potentially, help other algorithms [20,21,22,23,24] to improve the obtained results by automating the task of determining the patient’s basal needs and removing the need for initially establishing the basal profile for the therapy. 

## Figures and Tables

**Table 1:** Percentage of Time In Range-Batch 1.

	BG < 70	70 < BG < 180	BG > 180	BG > 250
Children	0%	100%	0%	0%
Adolescents	0%	100%	0%	0%
Adults	0%	100%	0%	0%

**Table 2:** Percentage of Time In Range-Batch 2.

	BG < 70	70 < BG < 180	BG > 180	BG > 250
Children	0%	82%	18%	6%
Adolescents	0%	92%	8%	1%
Adults	0%	96%	4%	0%

**Table 3:** Percentage of Time In Range-Batch 3.

	BG < 70	70 < BG < 180	BG > 180	BG > 250
Children	0%	100%	0%	0%
Adolescents	0%	100%	0%	0%
Adults	0%	100%	0%	0%

**Table 4:** Percentage of Time In Range-Batch 4.

	BG < 70	70 < BG < 180	BG > 180	BG > 250
Children	0%	79%	21%	6%
Adolescents	0%	91%	9%	2%
Adults	0%	96%	4%	0%

**Table 5:** Total Daily Dose (TDD) comparison between batches.

Patient	Simulation Batch 1			Simulation Batch 3				Increment
	TDD	TDD 0 to 12	TDD after 12	TDD	TDD 0 to 12	TDD after 12	BLOCKAGE	TDD from 12
adolescent#001	33.36	17.39	15.98	35.71	17.39	18.32	3.62	2.34
adolescent#002	29.62	14.78	14.84	30.80	14.78	16.03	3.62	1.18
adolescent#003	17.65	9.47	8.18	19.35	9.47	9.88	2.24	1.70
adolescent#004	25.64	13.47	12.17	28.04	13.47	14.57	3.02	2.41
adolescent#005	22.84	11.90	10.94	25.12	11.90	13.22	3.02	2.28
adolescent#006	31.78	15.57	16.20	34.03	15.57	18.46	3.62	2.26
adolescent#007	20.54	10.00	10.55	21.02	10.00	11.03	3.02	0.48
adolescent#008	20.09	9.79	10.30	20.44	9.79	10.65	3.02	0.35
adolescent#009	17.88	8.60	9.28	18.85	8.60	10.25	2.42	0.97
adolescent#010	21.35	11.04	10.32	23.24	11.04	12.21	2.90	1.89
adult#001	41.13	21.69	19.44	44.36	21.69	22.67	4.83	3.23
adult#002	45.42	23.67	21.75	48.44	23.67	24.77	4.83	3.02
adult#003	44.14	22.97	21.17	47.69	22.97	24.72	4.83	3.54
adult#004	26.49	13.88	12.61	28.78	13.88	14.90	3.02	2.29
adult#005	46.79	24.05	22.74	50.18	24.05	26.13	4.83	3.39
adult#006	44.53	24.87	19.66	49.00	24.87	24.13	6.04	4.47
adult#007	36.20	19.07	17.13	39.85	19.07	20.78	4.53	3.65
adult#008	35.98	18.78	17.19	38.58	18.78	19.80	3.62	2.60
adult#009	41.90	22.39	19.51	44.50	22.39	22.11	3.62	2.60
adult#010	43.22	22.42	20.80	45.87	22.42	23.45	3.62	2.65
child#001	10.22	5.32	4.90	11.67	5.32	6.35	1.81	1.45
child#002	10.37	5.32	5.06	11.72	5.32	6.40	1.45	1.34
child#003	7.67	3.96	3.71	8.49	3.96	4.54	0.97	0.83
child#004	12.19	6.31	5.89	14.32	6.31	8.01	2.42	2.13
child#005	13.85	6.99	6.86	15.08	6.99	8.09	1.63	1.23
child#006	11.58	5.97	5.61	12.72	5.97	6.75	1.39	1.14
child#007	13.90	6.92	6.98	14.81	6.92	7.89	1.51	0.91
child#008	10.81	5.73	5.08	11.84	5.73	6.11	1.51	1.02
child#009	11.10	5.76	5.34	12.95	5.76	7.19	2.42	1.85
child#010	11.71	5.97	5.74	12.72	5.97	6.74	1.45	1.00

## Data Availability

Available online: https://github.com/jberian/closed_loop/tree/master/Simulation/simglucose_with_blockage (accessed on 8 June 2021).
